# Genomic Analyses of Potential Novel Recombinant Human Adenovirus C in Brazil

**DOI:** 10.3390/v12050508

**Published:** 2020-05-04

**Authors:** Roozbeh Tahmasebi, Antonio Charlys da Costa, Kaelan Tardy, Rory J. Tinker, Flavio Augusto de Padua Milagres, Rafael Brustulin, Maria da Aparecida Rodrigues Teles, Rogério Togisaki das Chagas, Cassia Vitória de Deus Alves Soares, Aripuana Sakurada Aranha Watanabe, Cecilia Salete Alencar, Fabiola Villanova, Xutao Deng, Eric Delwart, Adriana Luchs, Élcio Leal, Ester Cerdeira Sabino

**Affiliations:** 1 Polytechnic School of University of Sao Paulo, Sao Paulo 01246-903, Brazil; sabinoec@gmail.com; 2 Institute of Tropical Medicine, University of Sao Paulo, Sao Paulo 01246-903, Brazil; kaelan.tardy@gmail.com; 3 Faculty of Biology, Medicine and Health, University of Manchester, Manchester M13 9PL, UK; rorytinker2011@gmail.com; 4 LIM/46, Faculty of Medicine, University of Sao Paulo, Sao Paulo 01246-903, Brazil; flaviomilagres@uft.edu.br (F.A.d.P.M.); eu3rafael@gmail.com (R.B.); 5 Secretary of Health of Tocantins, Tocantins 77453-000, Brazil; m.teles@yahoo.com.br (M.d.A.R.T.); chagastogisaki@hotmail.com (R.T.d.C.); cassiavitoriaalves@gmail.com (C.V.d.D.A.S.); 6 Institute of Biological Sciences, Federal University of Tocantins, Tocantins 77001-090, Brazil; 7 Public Health Laboratory of Tocantins State (LACEN/TO), Tocantins 77016-330, Brazil; 8 Department of Parasitology, Microbiology and Immunology, Federal University of Juiz de Fora, Juiz de Fora, MG 34092829, Brazil; almasurfe@yahoo.com.br; 9 Central Laboratory Division-DLC-HCSP, Clinical Laboratory and LIM 03-Department of Pathology, Clinical Hospital, University of Sao Paulo Medical School, Sao Paulo 01246-000, Brazil; cecialencc@gmail.com; 10 Institute of Biological Sciences, Federal University of Para, Para 66075-000, Brazil; fevface@gmail.com; 11 Vitalant Research Institute, 270 Masonic Avenue, San Francisco, CA 94118-4417, USA; xdeng@bloodsystems.org (X.D.); eric.delwart@ucsf.edu (E.D.); 12 Department Laboratory Medicine, University of California San Francisco, San Francisco, CA 94143, USA; 13 Enteric Disease Laboratory, Virology Center, Adolfo Lutz Institute, Sao Paulo 01246-000, Brazil; driluchs@gmail.com

**Keywords:** Adenovirus C, virome, recombination, gastroenteritis, phylogenetics

## Abstract

Human Adenovirus species C (HAdV-C) is the most common etiologic agent of respiratory disease. In the present study, we characterized the nearly full-length genome of one potential new HAdV-C recombinant strain constituted by Penton and Fiber proteins belonging to type 89 and a chimeric Hexon protein of types 1 and 89. By using viral metagenomics techniques, we screened out, in the states of Tocantins and Pará, Northern and North regions of Brazil, from 2010 to 2016, 251 fecal samples of children between 0.5 to 2.5 years old. These children were presenting acute diarrhea not associated with common pathogens (i.e., rotavirus, norovirus). We identified two HAdV-C strains in two distinct patients. Phylogenetic analysis performed using all complete genomes available at GenBank database indicated that one strain (HAdV-C BR-245) belonged to type 1. The phylogenetic analysis also indicated that the second strain (HAdV-C BR-211) was located at the base of the clade formed by the newly HAdV-C strains type 89. Recombination analysis revealed that strain HAdV-C BR-211 is a chimera in which the variable regions of *Hexon gene* combined HAdV-C1 and HAdV-C89 sequences. Therefore, HAdV-C BR-211 strain possesses a genomic backbone of type HAdV-C89 and a unique insertion of HAdV-C1 in the Hexon sequence. Recombination may play an important driving force in HAdV-C diversity and evolution. Studies employing complete genomic sequencing on circulating HAdV-C strains in Brazil are needed to understand the clinical significance of the presented data.

## 1. Introduction

Human Adenoviruses (HAdVs) are non-enveloped, double-stranded, medium-sized (34–36 kbp) linear DNA viruses classified in the genus *Mastadenovirus* [1,2]. The HAdV virion is icodsahedral in shape, made up of a 252-capsomer protein capsid, with 12 penton bases pentamers connected each to a fiber protein, and 240 hexon trimers [2]. There are currently seven known Adenovirus species that infect humans (HAdV-A to HAdV-G), divided into more than 100 distinct subgroups or subtypes (http://hadvwg.gmu.edu/). Recently, HAdV Working Group was established with the goal of standardizing the process of assigning names to candidate novel HAdVs. Nomenclature was developed to incorporate molecular types including major capsid genes penton base, hexon and fiber (PHF) (http://hadvwg.gmu.edu).

HAdVs are transmitted by multiple mechanisms including droplets, fomites, the fecal–oral route and autoinoculation. The infected host can develop a number of pulmonary complications including upper respiratory impairment, bronchiolitis and pneumonia, without significant seasonal variation [3]. The virus also causes extrapulmonary pathologies including conjunctivitis, gastroenteritis and meningitis [4,5]. Viruses of the HAdV-C species are universally prevalent and commonly associated with respiratory tract infections among pediatric patients [6]. After the initial primary infection, HAdV-C has the potential to remain in the lymphoid cells of the body and continue to intermittently shed viruses into the feces for many years [7].

Despite the global disease burden of HAdV-C and its use in molecular biology, only six HAdV-C types (HAdV-C1, HAdV-C2, HAdV-C5, HAdV-C6, HAdV-C57, and HAdV-C89) have been formally recognized so far [7,8]. This has resulted in a deficit of knowledge regarding the pathogenicity and evolution of HAdV-C [7]. HAdV-C recombinant viruses have been thoroughly investigated, allowing complex delineation of their genomes, resulting in the widespread use of HAdV in vector-based vaccines and gene therapies [8,9]. HAdV-C57 and HAdV-C89 are both recombinant viruses [8,10]. HAdV-C57 and HAdV-C6 share the same hexon structure and fiber gene [10]. In contrast, HAdV-C89 had a novel penton base sequence [7].

Full-genome sequencing has recently been used to investigate HAdV in fecal specimens from immunocompromised children [11,12]. The study demonstrated the widespread prevalence of HAdV-C strains in the British pediatric population [11]. The high prevalence of HAdV-C and the long intracellular life of its DNA in the host result in frequent superinfections, facilitating the evolution of novel HAdV-C types [11]. Here we performed genomic and bioinformatics analyses of one potentially new HAdV-C recombinant strain that was obtained from children with acute gastroenteritis in the Northern region of Brazil during a Next-Generation Sequencing (NGS) investigation of enteric viruses.

## 2. Materials and Methods

### 2.1. Study Population

The current cross-sectional surveillance study was carried out from 2010 to 2016 in the states of Tocantins and Pará, Northern and North regions of Brazil, respectively. This descriptive study was carried out with surveillance specimens from patients presenting with the symptoms of acute gastroenteritis at the Brazilian Unified Health System (SUS) units. A total of 251 fecal samples were collected for analysis from collaborating units in 38 different localities. Two hundred forty-five samples were collected from the state of Tocantins and 3 samples from the state of Pará. Three samples were obtained from border municipalities (Estreito and Carolina) located between the state of Tocantins and the state of Maranhão (Northeast region of Brazil). All specimens were sent to Tocantins Public Health Laboratory (LACEN-TO) together with relevant collection date, age and gender data. Fecal samples were screened for bacteria (e.g., *Escherichia coli* and *Salmonella sp.*), protozoan (e.g., *Giardia sp*.) and helminths (e.g., *Taenia Solum*) using conventional parasitological and cultures techniques. Stored frozen fecal specimens were then forwarded to the São Paulo Institute of Tropical Medicine to identify enteric viruses (e.g., rotavirus and norovirus) throughout NGS investigation. The specimens were stored at −20 °C.

Two HAdV-C strains were identified during the NGS investigation: HAdV-C BR-211 (potential novel HAdV-C recombinant) and HAdV-C BR-245 (HAdV-C1 strain), in which Norovirus was also detected (see the Figure S1 that shows all viruses found by NGS in these patients). Both patients were experiencing acute gastroenteritis symptoms, such as diarrhea, vomiting and fever. In addition, the HAdV-C BR-211 patient presented coryzal symptoms. HAdV-C BR-211 (potential novel HAdV-C recombinant) was detected in a sample collected in 2015 in the city of Carolina, Maranhão from a 3-year-old female child, and the HAdV-C BR-245 (HAdV-C1) was collected in 2014 in the city of Araguaína, Tocantins from a 1-year-old female infant.

### 2.2. Viral Metagenomics

The protocol used to perform deep sequencing was a combination of several protocols applied to viral metagenomics and/or virus discovery and has been previously described by the study of the current author [13]. In summary, 50 mg of human fecal sample was diluted in 500 μL of Hanks’ buffered salt solution (HBSS). This solution was then added to a 2 mL impact-resistant tube containing lysing matrix C (MP Biomedicals, Santa Ana, CA, USA) and homogenized in a FastPrep-24 5G Homogenizer (MP Biomedicals, Santa Clara, CA, USA). The homogenized sample was centrifuged at 12,000× *g* for 10 min, and approximately 300 μL of the supernatant was percolated through a 0.45 μm filter (Merck Millipore, Billerica, MA, USA) to remove eukaryotic- and bacterial-cell-sized particles. Approximately 100 μL, equivalent to one-fourth of the volume of the tube, of cold PEG-it Virus Precipitation Solution (System Biosciences, Palo Alto, CA, USA) was added to the filtrate, and the contents of the tube were gently mixed, then incubated at 4 °C for 24 h. After the incubation period, the mixture was centrifuged at 10,000× *g* for 30 min at 4 °C. Following centrifugation, the supernatant (~350 μL) was discarded. The pellet, rich in viral particles, was treated with a combination of nuclease enzymes (TURBO DNase and RNase Cocktail Enzyme Mix-Thermo Fischer Scientific, Waltham, MA, USA; Baseline-ZERO DNase-Epicentre, Madison, WI, USA; Benzonase-Darmstadt, Darmstadt, Germany; and RQ1 RNase-Free DNase and RNase A Solution-Promega, Madison, WI, USA) to digest unprotected nucleic acids. The resulting mixture was subsequently incubated at 37 °C for 2 h. After incubation, viral nucleic acids were extracted using a ZR & ZR-96 Viral DNA/RNA Kit (Zymo Research, Irvine, CA, USA) according to the manufacturer’s instructions. Subsequently, a Nextera XT Sample Preparation Kit (Illumina, San Diego, CA, USA) was used to construct a DNA library, which was identified using dual barcodes. The library was then purified using ProNex^®^ Size-Selective Purification System (Promega, WI, USA). Following ProNex^®^ purification, the quantity of each sample was normalized to ensure equal library representation in our pooled samples using the ProNex^®^ NGS Library Quant Kit (Promega, WI, USA). For size range selection, Pippin Prep (Sage Science, Inc.) was used to select a 300 bp insert (range 200–400 bp), which excluded very short and long library fragments. Prior to cluster generation, libraries were quantified again by qPCR using the ProNex^®^ NGS Library Quant Kit (Promega, WI, USA). The library was deep-sequenced using a Hi-Seq 2500 Sequencer (Illumina, CA, USA) with 126 bp ends [13,14,15]. Bioinformatics analysis was performed according to the protocol previously described [16]. The contigs, including sequences of rotaviruses as well as enteric viruses, humans, fungi, bacteria and others, sharing a percent nucleotide identity of 95% or less were assembled from the obtained sequence reads by de novo assembly. The resulting singlets and contigs were analyzed using BLASTx to search for similarity to viral proteins in GenBank. The contigs were compared to the GenBank non-redundant nucleotide and protein databases (BLASTn and BlastX). After identification of the viruses, a reference template sequence was used to map the full-length genome with Geneious R9 software (Biomatters Ltd. L2, 18 Shortland Street Auckland, 1010, New Zealand). Based on the best hits of the BLASTx searches, HAdV-C genomes were chosen for further analyses. Sequences generated in this study have been deposited in GenBank: MN628614 (HAdV-C BR-245) and MN628615 (HAdV-C BR-211). All protocols and procedures were conducted within the enhanced laboratory biosafety level 2 (ABSL-2) facility of the Institute for Tropical Medicine, São Paulo University. The ABSL-2 facility consists of a laboratory in which all in vitro experimental work is carried out in class 3 biosafety cabinets, which are also negative pressurized (<−200 Pa). Although all experiments are conducted in closed-class 3 cabinets and isolators, special personal protective equipment, including laboratory suits, gloves and FFP3 face-masks is used. Air released from the class 3 units is filtered by High-Efficiency Particulate Air (HEPA) filters and then leaves via the facility ventilation system, again via HEPA filters. Only authorized personnel that have received the appropriate training can access the facility. The facility is secured by procedures recognized as appropriate by the institutional biosafety officers and facility management at São Paulo University and Brazilian National Technical Biosafety Commission (CTNBio).

### 2.3. Phylogenetic Analysis

Genomes were aligned using Clustal X version 2.0 software [17]. Briefly, phylogenetic tree construction used the Maximum Likelihood approach, and branch support values were assessed with a Shimodaira-Hasegawa test. Trees were then inferred using FastTree version 2.1 software [18]. Evolutionary models were selected based on the likelihood ratio test (LRT) implemented in the jModeltest2 software [19]. Estimates of genetic distances were conducted using the Maximum Composite Likelihood model implemented in MEGA X version 10.0.5 [20]. All positions containing gaps and missing data were eliminated (complete deletion option). The number of base substitutions per site from between sequences is shown; standard error estimate(s) are also shown and were obtained by a bootstrap procedure (200 replicates). There was a total of 33,317 positions in the final dataset.

### 2.4. Detection of Recombination

The identification of potential parental sequences and the localization of possible recombination breakpoints were determined using the Recombination Detection Program, RDP4 version 4.9.5. [21], which was used with settings (a Bonferroni-corrected *p-*value cut-off of 0.05) for the different detection methods, including RDP5, GENECONV, BootScan, MaxChi, Chimaera, SiScan and 3Seq [21].

### 2.5. Homology Modeling of Hexon Structure

The primary amino acid sequence of HAdV-C BR-211 hexon was submitted to the SWISS-MODEL workspace for homology modeling [22]. The SWISS-MODEL template library (https://swissmodel.expasy.org) was searched with BLASTp and HHBlits [23] for evolutionary related structures that matched the target sequence [14,15,24]. The highest-quality templates were selected for model building. The models were built based on the target-template alignment using ProMod3. Coordinates that were conserved between the target and the template were copied from the template to the model. Insertions and deletions were remodeled by using a fragment library. Side chains were then rebuilt. PyMOL v0.99 (https:pymol.org/) was used to generate the space-filling representation of the hexon structure.

### 2.6. Ethical Approval

Previous Ethics Committee approval was granted by the University of São Paulo School of Medicine (number CAAE: 53153916.7.0000.0065) and Lutheran Palms University Center (number CAAE: 53153916.7.3007.5516). This was an anonymous unlinked study, and informed consent was not required according to resolution 466/12 concerning research involving humans (Conselho Nacional de Saúde/Ministério da Saúde, Brasília, 2012).

## 3. Results

### 3.1. Genetic Distances of Brazilian HAdV-C

Viral sequences were identified through sequence identity (using BLAST) to annotated viral genomes in GenBank. Near full-length genomes were used to estimate the evolutionary distances of the strains detected here to HAdV-C reference strains. Once the mean genetic distances within each type were less than 10%, one reference sequence per type was selected in order to calculate pairwise distances between Brazilian and reference strains. The highest distance to strain HAdV-C BR-211 was with type 5 (43%), and the lowest was with type 57 (less than 4%). Equally, the highest distance to strain HAdV-C BR-245 was with type 5 (68%) and the lowest with type 1 (29%). The distances between HAdV-C BR-211 and HAdV-C BR-245 were 65%. It is important to mention that the higher distances of HAdV-C BR-245 are partially due to the poor quality of these sequences, which have many ambiguities and multiple gaps. To provide more detailed estimates and avoid errors of genome regions with poor quality, we also estimated distances between penton base, hexon and fiber genes separately (Table 1). Based on the closest evolutionary distances (Boldface words in Table 1), HAdV-C BR-245 can be classified as P1H1F1 and BR-211 classified as P89H1F89. Overall, genetic distances are in consensus with Blast results. Phylogenetic analysis confirms that strain BR-245 can be classified as HAdV-C1 and that strain HAdV-C BR-211 is possibly a recombinant strain.

### 3.2. Phylogenetic Analysis of Near Full-Length Genomes of HAdV-C

**Figure 1:**
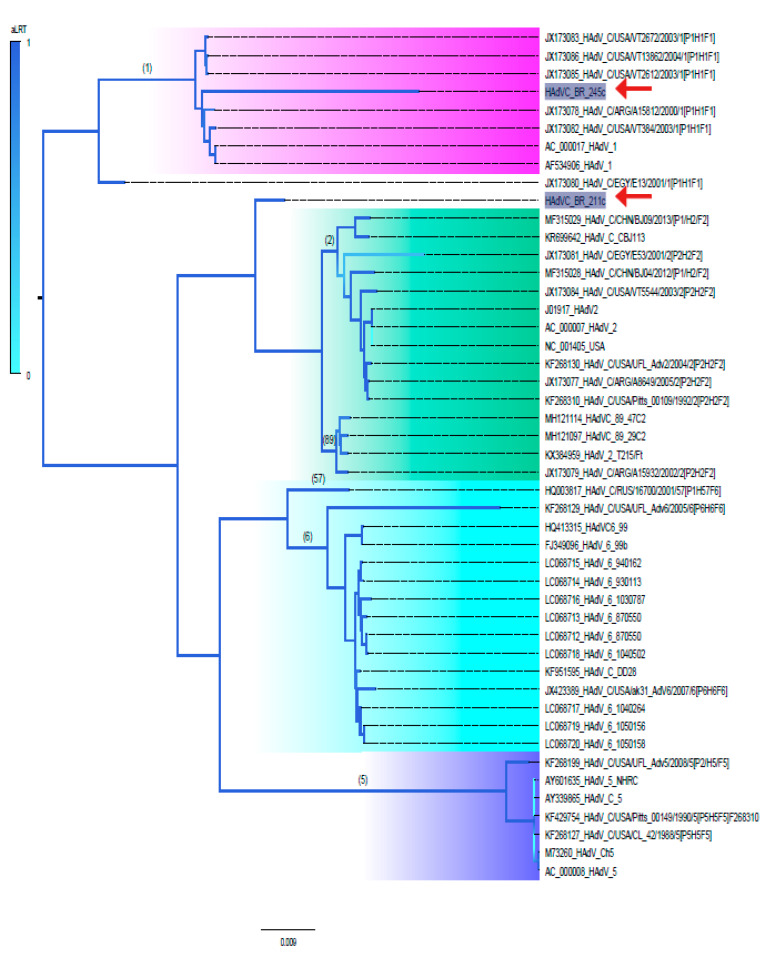
Maximum Likelihood tree constructed using near full-length genomes of Human Adenovirus species C (HAdV-C). Red arrows point to the Brazilian strains described in the present study. Each node and corresponding branches are colored according to their statistical likelihood, calculated using aLRT. Numbers above branches of each phylogroup correspond to HAdV-C types, and the overarching clades they belong to are highlighted in different colors. The scale bar under the tree represents nucleotide substitutions per site. The Maximum Likelihood tree was inferred assuming General time reversible (GTR) + Gamma correction model and was constructed using the software FastTree [18].

The Maximum Likelihood tree shown in Figure 1 was inferred using all near full-length genomes of HAdV-C available. Sequences formed well-supported monophyletic groups, represented by their corresponding prototype: types 1, 2, 5, 6, 57 and 89. Based on the phylogenetic tree, the HAdV-C BR-245 detected here grouped into the HAdV-C type 1 as expected. On the other hand, HAdV-C BR-211 strain was placed at the base of the clade formed by HAdV-C2 and HAdV-C89. It is important to mention that HAdV-C89 has been recently classified as a consequence of multiple recombination events [1,8,12].

**Figure 2:**
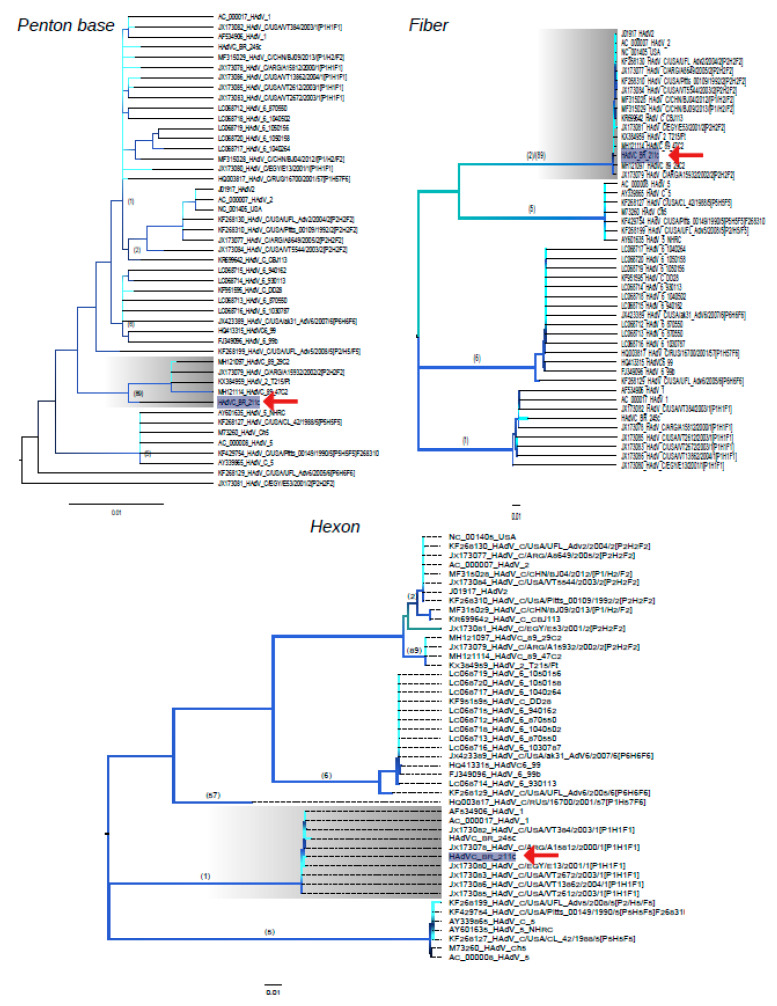
Maximum Likelihood trees comparing penton base, hexon and fiber regions of sample and reference HAdV-C strains. A red arrow points to the Brazilian strain HAdV-C BR-211. Numbers above branches of each phylogroup correspond to HAdV-C types. Phyloclades in which the Brazilian strain HAdV-C BR-211 belongs are highlighted in grey. Each node and corresponding branches are colored according to their statistical likelihood, calculated using aLRT. The scale bar under the trees represents nucleotide substitutions per site. Maximum Likelihood trees were inferred using the most likelihood model according to the aLRT. All trees were constructed using the software FastTree [18].

In order to classify HAdV-C BR-211, the three coding regions (penton base, hexon and fiber) were analyzed separately. Each region was used to infer phylogenetic relationships between HAdV-C BR-211 and reference strains. All genetic trees displayed high bootstrap values based on approximate LRT. The phylogenetic analysis of the partitioned regions confirmed that strain HAdV-C BR-211 exhibited a close genetic relationship to HAdV-C-89 strains in its penton base and fiber regions, while the hexon gene grouped into the HadV-C type 1 (Figure 2).

### 3.3. Recombination of HAdV-C1 and HAdV-C89

**Figure 3:**
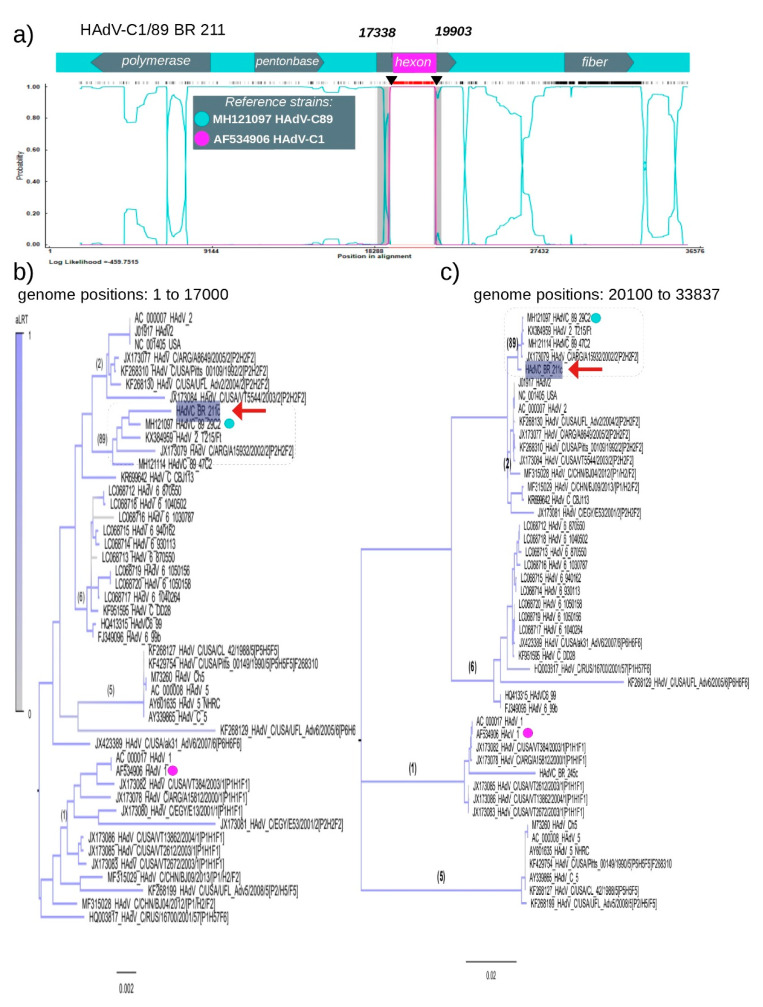
Recombination pattern of chimera strain HAdV-C BR-211. (**a**) The Burt method was used to determine the parental types that compose the recombinant HAdV-C BR-211 strain. Colored lines represent the probability (given by the hidden Markov model) of genomic regions belonging to a certain parental HAdV-C type (greenish-blue line—MH121097 HadV-C type 89—and magenta line—AF534906 HAdV-C type 1). The *x* axis represents the sequence length in base pairs (bp). The *y* axis represents the probability at each base. The diagram above the plot indicates the position and direction of the polymerase, penton base, hexon and fiber orfs on the genome of HAdV-C, and the vertical lines indicate the location breakpoints in the hexon region. Only results with a probability above 0.95 were considered. In the upper region of the figure, a hatched horizontal line represents the informative genome site used to determine recombination, and the red area is the interval of breakpoints. The confidence interval of breakpoints is indicated by vertical gray lines. All these analyses were performed using the RDP v4 software [21]. Maximum Likelihood trees were constructed with an alignment corresponding to the genome positions 1 to 17,000 (**b**) and with alignment corresponding to positions 20,100 to 33,837 (**c**). The Brazilian strain HAdV-C BR-211 is indicated by red arrows, and references are indicated by dots. The scale bar under the tree represents the nucleotide substitutions per site. Maximum Likelihood trees were inferred using the most likelihood model according to the aLRT. All trees were constructed using the software FastTree [18].

Recombination analysis of HAdV-C BR-211 strain (supposed P89H1F89 recombination) revealed a unique and unexpected feature: the presence of a potential chimeric hexon gene derived from HAdV-C1 and HAdV-C89. Two breakpoints located at positions 17,338 and 19,903 in the HAdV-C BR-211 genome were identified. The region delimited by the breakpoints is related to HAdV-C1 and was mapped within the hexon gene sequence, starting at position 17,186 and ending at position 20080. Hence, the HAdV-C BR-211 strain possesses a genomic backbone of type HAdV-C89 with a unique insertion of HAdV-C1 in the hexon sequence. The mosaic pattern of HAdV-C BR-211 is shown in Panel A in Figure 3, highlighting its composition of DNA from two distinct HAdV-C strains (represented by colored lines). To support our findings, the hexon gene sequence was partitioned in two: the first partition corresponds to nucleotides 1 to 17,000, and the second partition corresponds to the nucleotides 20,100 to 33,837, excluding the supposed HAdV-C1 insertion. Maximum Likelihood trees were constructed for each partition and confirmed that HAdV-C BR-211 belongs to type 89 in both regions (Panels B and C in Figure 3). We also constructed a genome tree without the hexon gene region (Figure S2) and characterized mosaic genome of BR-211 by other methods (Figure S3).

### 3.4. Structure of Recombinant Hexon Protein of HAdV-C

**Figure 4:**
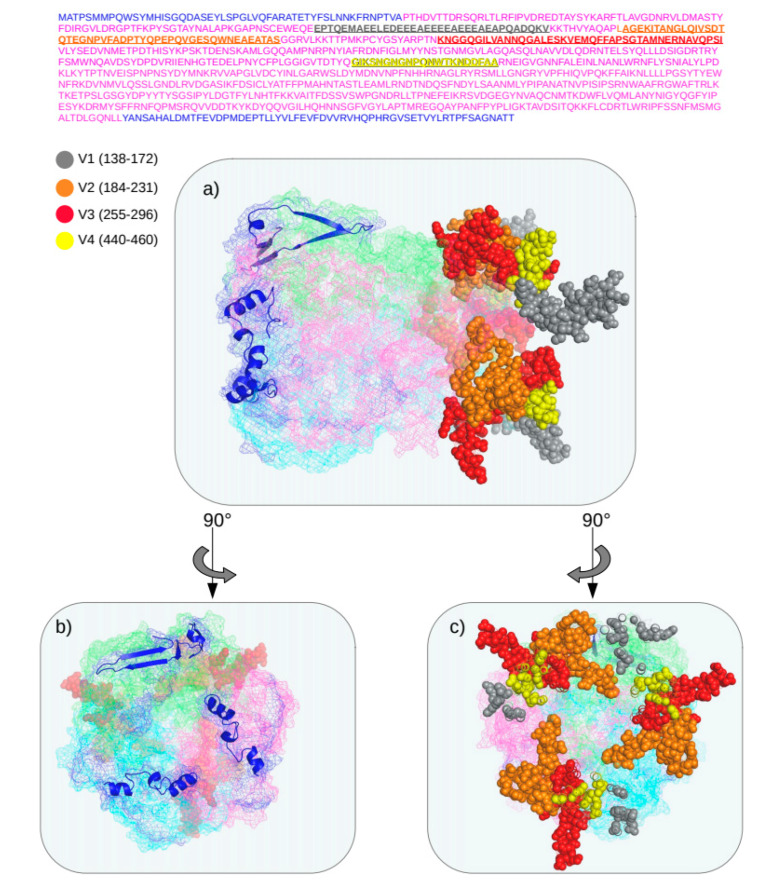
Homotrimeric structure of chimeric hexon protein of HAdV-C BR-211 strain. The predicted amino acid sequence of the hexon protein of the HAdV-C BR-211 strain is shown in the **upper panel** of the image. Residues depicted in blue are those related to HAdV-C89, and residues in magenta are those related to HadV-C1. Variable regions, related to HAdV-C1, and present on the apical side of the protein, are indicated in the following colors: gray (V1: residues 138–172), orange (V2: residues 184–231), red (V3: residues 255–296) and yellow (V4: residues 440–460). (**a**) Side view of the predicted 3D model of the chimeric hexon protein of the HAdV-C BR-211 strain based on the crystal structure of HAdV-C2 (PDB; 1P2Z). Mesh representation of each monomer of the hexon protein are distinguished by color: cyan, magenta and light green. Blue structures in the 3D model highlight regions of the HAdV-C BR-211 hexon protein related to HAdV-C89. Variable regions (V1, V2, V3 and V4) of the hexon protein are shown as colored spheres in the 3D model. (**b**) and (**c**) show, respectively, left- and right-sided views of the 3D hexon model. The homology model was based on the highest BLASTp scores and was made using Swiss-model tools [22,23].

We were interested in understanding whether there were differences in the conformation of a chimeric hexon, so the structure of the hexon of the HAdV-C BR-211 chimera was investigated. Based on the high BLASTp similarity score between the amino acid sequences of the hexon proteins of HAdV-C BR-211 and HAdV-C2, the hexon protein structure 1P2Z from a HAdV-C2 strain was selected as a template to predict the structure of the HAdV-C BR-211 chimeric hexon. The HAdV-C BR-211 sequence was mapped onto the HAdV-C2 structure. Of note, the variable regions present on the apical side of the hexon proteins, facing the outside of the virion, are derived from HAdV-C1, but remain unique (Figure 4). Variable regions of each monomer are interlaced to form the trimeric structure.

### 3.5. Characteristics of Penton Base of HAdV-C BR-211

Trees constructed with the nucleotide or amino acid sequences of the penton base gene showed that the HAdV-C BR-211 strain forms a clade with HAdV-C89. Recombination analysis did not reveal any breakpoints in this region. Recently, HAdV-C89 was characterized as a new HAdV-C type, and this was achieved based on penton base residue analysis [7]. After initial viral attachment, the penton base interacts with cellular integrins through an Arg–Gly–Asp (RGD) motif located in a hypervariable surface loop. This process results in virus internalization [25]. It is with this hypervariable surface loop that penton base proteins can be distinguished. Key residues of the surface loop used to distinguish HAdV-C89 from HAdV-C2 [8] were used here to differentiate HAdV-C BR-211 from the other HAdV-C types. Predicted amino acid sequences of HAdV-C BR-211 penton base were compared to the amino acid sequences of reference strains. Table 2 summarizes these findings. Of the key residues on the hypervariable surface loop, HAdV-C BR-211 shares all the same residues as HAdV-C89 except for an amino acid substitution in strain HAdV-C BR-211 at position 157 (K→N). In addition, motif ^361^AAAP^364^, present in HAdV-C BR-211 and other HAdV-C types like type 2, is absent in HAdV-C89. These features, which distinguish HAdV-C BR-211 from HAdV-C89, are in boldface in Table 2.

## 4. Discussion

In this study, 251 fecal samples from children with acute gastroenteritis were surveyed using NGS. Detailed descriptions of viruses detected in these samples were reported elsewhere [14,15,24,26,27,28,29,30,31,32]. Here, we characterized the near full-length genomes of two Brazilian HAdV-C strains. The detection of HAdV-C strains in stool samples could not necessarily be associated with diarrhea symptoms, as HAdV can exhibit a lingering shedding in feces after previous infections in other organs [33]. In fact, HAdV-C1, to which the HAdV-C BR-245 strain is identified here, is frequently associated with respiratory infections [34]. In addition, patient HAdV-C BR-245 tested positive for Norovirus infection. Patient HAdV-C BR-211 was experiencing respiratory symptoms in addition to gastroenteritis.

Previous studies have reported the detection of HAdV-C1 strains in Brazil associated with both respiratory and gastrointestinal symptoms [34,35]. Nevertheless, to the best of our knowledge, this is the first description of the full-length genome of a recombinant HAdV-C strain (BR-211) in the country. The HAdV-C1 BR-245 strain revealed to be related to Argentinean and North American HAdV-C1 strains according to our analysis, suggesting that this particular HAdV-C1 strain may have been circulating in the North and South American continents. However, the dataset used for this analysis presented an important limitation: the number of sequences analyzed was small, a restriction mainly due to the fact that we chose to look at only complete or near full-length genome sequences. More in-depth analyses of HAdV-C1 strains detected worldwide involving full genomic characterization are needed in order to expand our understanding of HAdV-C1 genetic diversity.

NGS surveillance, described in the present investigation to study enteric viruses, has provided an opportunity to identify for the first time in Brazil a strain, BR-211, belonging to a new HAdV-C type derived from the recently described HAdV-C type 89. Because HAdV-C89 was only recently described as a novel HAdV-C strain [7], knowledge of the epidemiology and prevalence of HAdV-C89 is still very limited. This new HAdV-C type could only be determined based on the sequencing of its entire genome, since approximately 80% of the viral sequence shares high similarity to HAdV-C2 strains [7]. Hence, without analyzing the whole genome of BR-211, it would have been virtually impossible to confirm its type and origins. Recombination events between the genes encoding major capsid proteins (hexon, fiber and penton base) are known to play an important role in the evolution of HAdV-D types [35], but members of the HAdV-C species are considered to be more stable, undergoing fewer recombination events [7,36]. HAdV-C types 57 and 89 are exceptions to this rule [7,9]. The present study also identified, in HAdV-C BR-211, a chimeric hexon protein combining type 1 and type 89 sequences. Moreover, the variable regions in the hexon protein of HAdV-C BR-211 were found to be related to HAdV-C type 1 and not type 89. The variable regions of the HAdV hexon proteins represent the major antigenic portion of the protein, and various neutralizing antibodies have been detected that target them [37,38,39]. It also has been shown that site-directed mutations in the variable regions of hexon proteins reduce drastically the replication of HAdV-C [39].

Characterizing recombination events allow one to indirectly identify HAdV-C strains that are circulating at a certain location and at a certain time. In other words, recombination means co-infection and therefore co-circulation of different strains [12]. Our NGS data is not enough to determine the source and HAdV-C BR-211 infection. This strain may have emerged recently by the recombination of strains HAdV-C1 and HAdV-C89 that were co-infecting the patient BR-211. Another equally likely explanation is that patient 211 was infected by a HadV-C1/89 recombinant strain. For this reason, an increase in HAdV-C surveillance in this particular area of Brazil could provide valuable information about the presence and prevalence of strains HAdV-C1 and HadV-C89 and other recombinant HAdV-C strains.

In addition to the chimeric hexon with type 1 and type 89 sequences identified in HAdV-C BR-211, we also found unique signatures in the penton base (Table 2). The HAdV-C89 penton base sequence is highly diverse in the functional RGD loop compared to the HAdV-C types described in the past, which may result in differences in the binding properties of type 89 to secondary cellular receptors. However, whether such amino acid differences in penton base sequences might increase or not the virulence of circulating HAdV-C89 strains has yet to be assessed.

## 5. Conclusions

Here we describe a near full-length genome of two HAdV-C strains from Brazil: the BR-245 strain, which is a common HAdV-C type 1 strain, and the HAdV-C BR-211 strain, a potential new recombinant HAdV-C strain with its own type, made up in part by a chimeric hexon gene with type 1 and type 89 DNA. Routine HAdV-C typing strategies may fail to detect new recombinants, likely resulting in incorrect typing of novel HAdV-C strains and missing the opportunity to discover new HAdV-C recombinants or even entirely new types. Expanding the viral genome database could improve overall typing success and help track the emergence of novel HAdV-C strains, thus helping to understand their evolution. In addition, future studies employing complete genomic sequencing on circulating HAdV-C strains in Brazil are needed to understand the clinical significance of the data presented in this study.

## Figures and Tables

**Table 1:** Genetic divergence of HAdV-C BR-211 and HAdV-C BR-245 strains.

Gene Region	Type 1	Type 2	Type 5	Type 6	Type 57	Type 89	Strain
Penton base (P)	0.00886	0.01065	0.01424	0.00886	0.00896	**0.00510**	BR-211
	**0.00235**	0.00886	0.01663	0.00471	0.00335	0.01543	BR-245
Hexon (H)	**0.00242**	0.16695	0.20007	0.19355	0.13400	0.17017	BR-211
	**0.00446**	0.017060	0.20023	0.19549	0.14343	0.17385	BR-245
Fiber (F)	0.777510	0.004093	0.722381	0.685276	0.692742	**0.000994**	BR-211
	**0.025583**	0.775442	0.691595	0.598394	0.605335	0.779578	BR-245

Genetic distances were calculated using Maximum composite likelihood implemented in Mega X. Those based on the closest evolutionary distances are in boldface.

**Table 2:** Differences at the variable region of penton base of HAdV-C strains.

Penton Base Position	HAdV-C				
	BR-211	Type 89	Type 2	Type 5	Type 1
2	R	R	Q	R	R
153	Q	Q	L	P	L
157	**K**	**N**	K	N	K
312	S	S	N	S	N
458	R	R	S	R	R/S
361–364	**AAAP**	**Del.**	AAAP	AAAP	AAAP
367–369	EAA	EAA	Del.	Del.	EAA

IUPAC amino acid code. A = Alanine, C = Cysteine, D = Aspartic Acid, E = Glutamic Acid, F = Phenylalanine, G = Glycine, H = Histidine, I = Isoleucine, K = Lysine, L = Leucine, M = Methionine, N = Asparagine, P = Proline, Q = Glutamine, R = Arginine, S = Serine, T = Threonine, V = Valine, W = Tryptophan, Y = Tyrosine. Del. = Deletion. These features, which distinguish HAdV-C BR-211 from HAdV-C89, are in boldface.

## Supplementary Materials

The following are available online at https://www.mdpi.com/1999-4915/12/5/508/s1, Figure S1: viruses identified by metagenomics, Figure S2: phylogenetic tree of HAdV-C genomes and Figure S3: genome mosaic pattern of HAdV-C 211.
