# Supplementary material for: Genomic Analyses of Potential Novel Recombinant Human Adenovirus C in Brazil
Source: Viruses. 2020 May 4;12(5):508. doi: 10.3390/v12050508 (PMC7290489; doi:10.3390/v12050508)
Supplement: Supplementary file 1 [file viruses-12-00508-s001.pdf]

Supplementary material:

Genomic Analyses of Potential Novel Recombinant Human Adenovirus C in Brazil

Roozbeh Tahmasebi[1,2,*,†], Antonio Charlys da Costa[2,*,†], Kaelan Tardy[2], Rory J Tinker[3], Flavio Augusto de Padua Milagres[4,5,6,7], Rafael Brustulin[4,5,6], Maria da Aparecida Rodrigues Teles[5,7], Rogério Togisaki das Chagas[5,7], Cassia Vitória de Deus Alves Soares[5,7], Aripuana Sakurada Aranha Watanabe[8], Cecilia Salete Alencar[9], Fabiola Villanova[10], Xutao Deng[11,12], Eric Delwart[11,12,§] Adriana Luchs[13,§] Élcio Leal[10,§,†], Ester Cerdeira Sabino[1,2,4,§]

1Polytechnic School of University of Sao Paulo, Sao Paulo, Brazil; roozbeh@usp.br (R.T.)
2Institute of Tropical Medicine, University of Sao Paulo, Sao Paulo, Brazil; sabinoec@gmail.com (E.C.S.); charlysbr@yahoo.com.br (A.C.d.C); kaelan.tardy@gmail.com (K.T.)
3Faculty of Biology, Medicine and Health, University of Manchester, Manchester, M13 9PL, UK; rorytinker2011@gmail.com (R.J.T.)
4LIM/46, Faculty of Medicine, University of Sao Paulo, Sao Paulo 01246-903, Brazil; flaviomilagres@uft.edu.br (F.A.d.P.M) sabinoec@gmail.com (E.C.S.);
5Secretary of Health of Tocantins, Tocantins, Brazil; eu3rafael@gmail.com (R.B.); chagastogisaki@hotmail.com (R.T.d.C.); cassiavitoriaalves@gmail.com (C.V.d.D.A.S.); m.teles@yahoo.com.br (M.A.R.T)
6Institute of Biological Sciences, Federal University of Tocantins, Tocantins, Brazil.
7Public Health Laboratory of Tocantins State (LACEN/TO), Tocantins, Brazil
8Department of Parasitology, Microbiology and Immunology, Federal University of Juiz de Fora, Juiz de Fora, MG, Brazil; almasurfe@yahoo.com.br (A.S.A.W.)
9Central Laboratory Division- DLC-HCSP, Clinical Laboratory and LIM 03-Department of Pathology, Clinical Hospital, University of Sao Paulo Medical School, Sao Paulo, Brazil; cecialencc@gmail.com (C.S.A.)
10Institute of Biological Sciences, Federal University of Para, Para 66075-000, Brazil; elcioleal@gmail.com (E.L.); fevface@gmail.com (F.V.)
11Vitalant Research Institute, 270 Masonic Avenue, San Francisco, CA 94118-4417, USA; xdeng@bloodsystems.org (X.D.); eric.delwart@ucsf.edu (E.D)
12Department Laboratory Medicine, University of California San Francisco, San Francisco, 94143 CA, USA;
13Enteric Disease Laboratory, Virology Center, Adolfo Lutz Institute, Sao Paulo 01246-000, Brazil; driluchs@gmail.com (A.L.)
*These authors contributed equally to this work
§These authors jointly supervised this work
†Correspondence: elcioleal@gmail.com (É.L.), charlysbr@yahoo.com.br (A.C.d.C.), roozbeh@usp.br (R.T)

a)

Patient 245

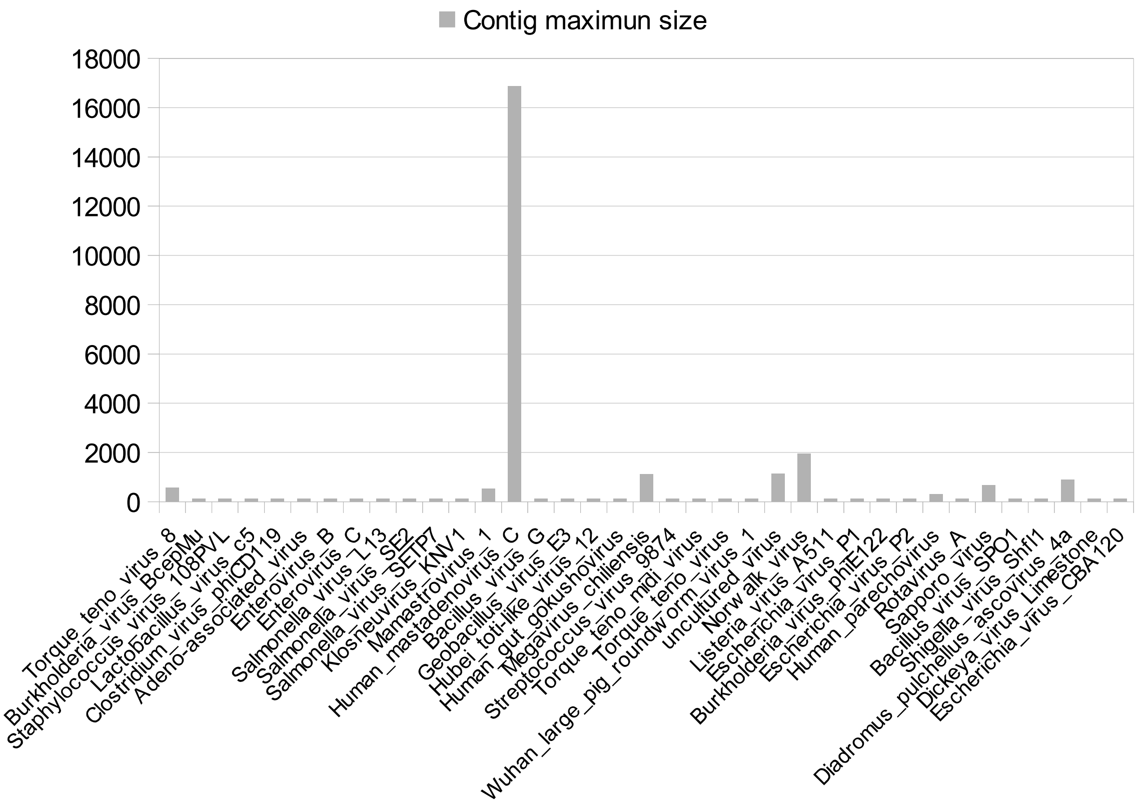

Patient 211

b)

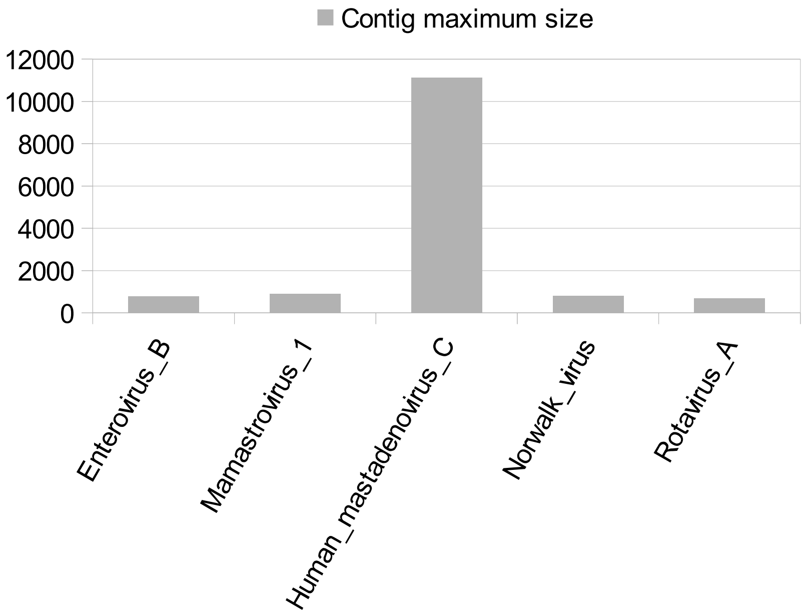

Figure S1. Viruses identified by metagenomic analysis
Metagenomic analysis of viruses in children with gastroenteritis. Diagram showing all viruses identified in a certain biological sample. X-axis represent viral species identified in each sample and y-axis represent the number of reads per species. A) viruses identified in the patient BR 211 and B) viruses identified in the patient BR 211

Genome tree (hexon region removed)

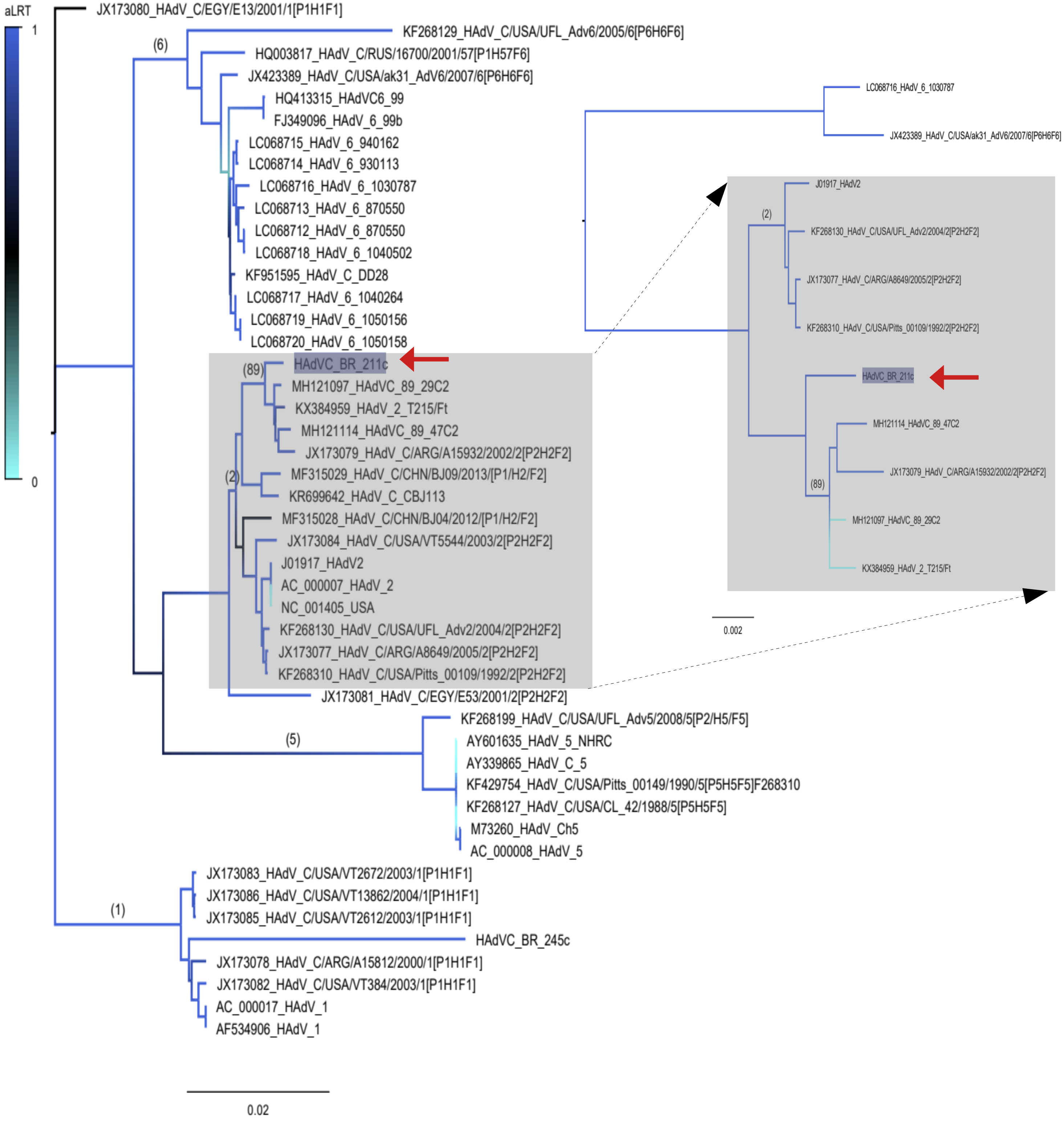

Figure S2. Phylogenetic tree of HAdV-C genomes
Maximum likelihood tree constructed using near-full length genome of HAdV-C in which the hexon gene region was exclude. The Brazilian strain BR211 described in the present study is indicated in the tree by red arrow. A colored scale indicating the statistical support of each node, calculated using aLRT, is shown in the tree. Phylogenetic groups corresponding to main genotypes are indicated by the numbers above branches of each phylogroup. The scale bar under the tree represents the nucleotide substitutions per site. A subtree constructed with few strains of types 2, 89 and 6 is also shown in the image. Maximum likelihood tree was inferred assuming GTR+gamma model and was constructed using the software FastTree v2.

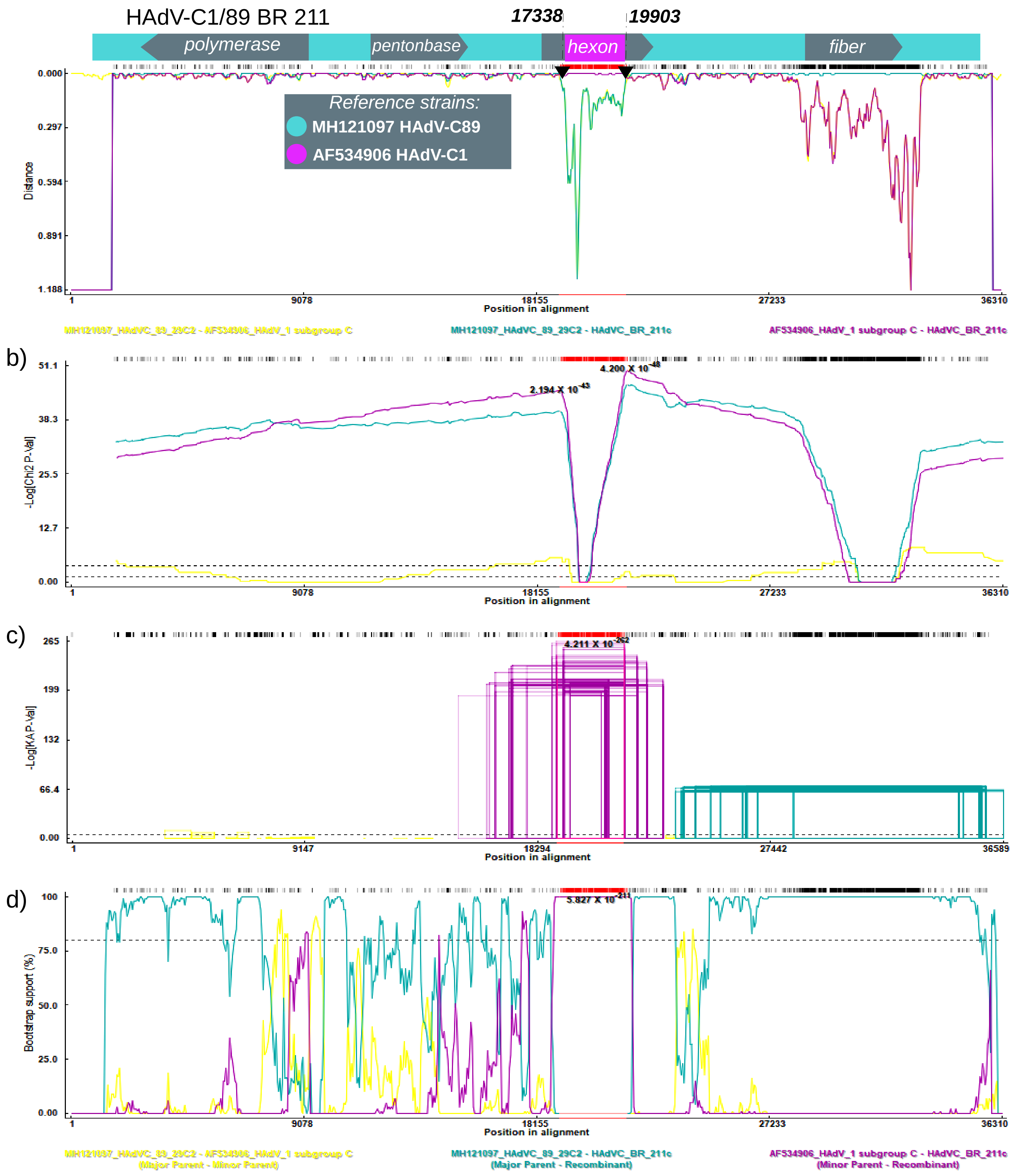

Figure S3. Genome mosaic pattern of HAdV-C 211
Recombination pattern of chimera strains HAdV-C BR 211. The mosaic pattern of BR 211 is shown using disticnt recombination methods that were described in material and methods section. A) The distance plot method. B) MaxChi method. C) Genconv method. D) Bootscanning method. Colored lines represent the probability (given in hidden Markov model approach) of genomic regions belonging to a certain parental HAdV-C type in this case type 89 (greenish blue line) and type 1 (magenta line). The x-axis represents the sequence length in base pairs (bp). The y-axis represents the statistical support in probability higher than 0.95. In the upper region of the figure a hatched horizontal line represent informative genome site used to determine recombination, red area is the interval of breakpoints. The confidence interval of breakpoints are indicated by vertical gray lines. All these analyses were performed using the RDP v4 software.
